# What Do the Dental Students Know about Infection Control? A Cross-Sectional Study in a Teaching Hospital, Rawalpindi, Pakistan

**DOI:** 10.1155/2020/3413087

**Published:** 2020-06-01

**Authors:** Mariam Khan Qamar, Babar Tasneem Shaikh, Aamir Afzal

**Affiliations:** ^1^Department of Pathology, Foundation University Medical College, Rawalpindi, Pakistan; ^2^Health Services Academy, Islamabad, Pakistan; ^3^Foundation University Medical College, Rawalpindi, Pakistan

## Abstract

**Background:**

Dental students are exposed to various infections and infective sources during their training, and on this aspect, their level of knowledge is suboptimal and practices are risky. Therefore, improving their knowledge and practices would contribute significantly to infection control.

**Objective:**

To ascertain the level of understanding of senior dental students regarding the infection control in the dental practice.

**Methods:**

A cross-sectional study was conducted among dental students (3rd year and 4th year) of the Foundation University Dental College, Pakistan. The sample consisted of 100 third year dental students and 88 fourth year students. A self-administrated questionnaire was used for data collection which consisted of fourteen close-ended items. Frequencies of knowledge, attitudes, and practice were calculated separately by using SPSS 21.0 software.

**Results:**

Almost half of the students would not use any antiseptic for sterilizing their hands, and only two-third would ask their patient to use an oral mouth rinse before starting the treatment. Many students did not the optimal temperature of the autoclave for sterilization of the instruments. Only one-third would wear the personal protective equipment during a procedure. Around one-third of the study participants reported that ineffective sterilization during clinical practice can transmit infection from one patient to another.

**Conclusion:**

Knowledge on infection control among the dental students is though weak, practices are not as per standards but attitudes are positive and encouraging for taking steps and complying with measures on infection control.

## 1. Background

Dental students are one of the dental health care professionals who are at a high risk of exposure to infections because of their direct contact with the patients, infected instruments, and hospital environment. Cross infection is a major concern to all the dental health care professionals. It is defined as the transmission of infection between the staff and the patient within the hospital environment [1]. Among these, the most serious oral infections are caused by bacteria that colonize in the oral cavity including mycobacterium tuberculosis, influenza virus, and streptococci. Students are equally vulnerable to cross infections that are caused by the hepatitis B virus (HBV), hepatitis C virus (HCV), and other viruses [2]. Moreover, they are also at a risk of percutaneous occupational injuries and eye exposure while treating the patients [3]. Only through strict safety precautions and implementation of guidelines for infection control, we can prevent these mishaps from happening. The Center for Disease Control and Prevention (CDC) in the United States has updated its guidelines on infection control in dental settings. The aim of these guidelines is to ensure a safe working environment to prevent the transmission of nosocomial and occupational infections among the dental health professionals [4, 5].

Dental colleges are responsible for providing proper training to their students to ensure safety of the patients, implementing infection control measures, and establishing a safe working environment [6]. Several studies have been conducted to assess the practices and knowledge of dental students and have demonstrated poor compliance of the students to infection control measures. A study conducted in India to assess the infection control practices among dental students showed that only one-tenth of the respondents adhere to the infection control measures [1]. Similar studies have been conducted worldwide to investigate the knowledge and practices of dental students on infection control [6–14], and a general consensus is that students need awareness and must be protected in the unsafe environment.

Fauji Foundation Hospital in Rawalpindi city houses a huge number of medical and dental students for educational and training purposes. This hospital is a busy tertiary health facility catering to mostly ex-army servicemen and their families. It has a fully equipped dental outdoor clinic where the dental undergraduate students come on daily basis for observations, education, hands-on training, or apprenticeship. Nevertheless, there is paucity of data with regard to infection control practices among dental students within the hospital environment in Pakistan. For this purpose, it is imperative to ascertain the understanding of the senior dental students who have attended the module of infection prevention and control during their study course. This study will help better understand the gaps and deficiencies in the dental college curriculum and will sensitize and educate the future dental surgeons in adopting the necessary infection prevention practices.

## 2. Methods

A cross-sectional study was conducted among dental students (3^rd^ year and 4^th^ year) attending the Foundation University Dental College, Pakistan. The duration of study was two months from 14^th^ July 2019 to 14^th^ September 2019. The sample size (*n* = 188) was calculated by using the WHO sample size calculator with the following parameters: confidence level = 95%, anticipated population proportion = 95.5% [6], and absolute precision required = 3%. A total of 228 dental students studying in the college included 121 third year and 107 fourth year students. Out of these approaches, 82.6% (*n* = 100) students from the third year and 82.2% (*n* = 88) students from the fourth year gave a complete response and therefore were included in the study. As per the exclusion criteria, the students of first and second year were excluded from the study because training in infection control is provided in the dentistry curriculum of third year and fourth year. Written informed consent was taken from all the participants of the study. The Institutional Review Board of the hospital granted the requisite permission to conduct the study. A total of 188 dental students completed the questionnaire comprising of 14 items. There were 6 questions related to “knowledge,” 5 questions related to practice, and 3 questions were based on “attitude.” A self-administered questionnaire developed by Singh et al. [6] was distributed to the dental students. The consistency of the questionnaire was assessed using Cronbach's alpha (*α* = 381). Each student was given fifteen minutes to fill the questionnaire in silence without discussing with each other. They were not asked to put their names on the forms, hence keeping the anonymity intact. Data was entered and analyzed on SPSS v.21. Descriptive statistics was done in terms of mean and standard deviation. Frequency and percentage were calculated for qualitative variables. Independent sample *t*-test was used to compare the mean score of knowledge, attitude, and practice among both groups. *P* value ≤ 0.05 was taken as a level of significance. Written verbal consent was obtained from all the respondents. Study received approval from the Ethics and Research Committee of the Foundation University, Rawalpindi, dated January 2, 2019.

## 3. Results

A total of 188 dental students responded to our questionnaire completely and hence included in the study results. Hence, the study population comprised of 28 male and 168 female students.  Table 1 shows the analysis of cross tabs between the independent (gender and class of study) and dependent variables which yielded no significant statistical association (i.e., *P* > 0.05).

Table 2 shows that although a majority (94%) reported washing their hands before and after examination of the patient, yet only half of them (49%) would use an antiseptic solution for washing their hands. There were 64% participants who preferred patients to have an oral mouth rinse before commencement of any treatment procedure. The majority (98%) of the students considered isolation as an important measure for the infection control. With regard to vaccination, 85% got vaccinated for hepatitis B, 45% for tetanus, and only 15% of the students were vaccinated for tuberculosis, but surprisingly, 18% have not received any vaccine at all.

On the subject of sterilization of instruments, 95% reported use of autoclave for the purpose and only 68% reported using the same for 15 minutes (optimal time) and only 60% thought that 120 degrees is the required temperature on which the autoclave should be used. Around 80% study participants considered that hepatitis B has the highest rate of transmission via saliva. In case of direct blood contact with a HIV patient, 62% would opt for anti-HIV immunoglobulins. During the work at a dental surgery, the use of face mask and gloves as an infection control measure was practiced by 38% while 32% would wear an eye protector, and only one-third of them (29%) would wear all of them. After using a pair of gloves on the patient, 57% would dispose them off but 43% think that they can reuse them after washing. Only 37% reported that ineffective sterilization during clinical practice can transmit infection from one patient to another, although a large majority (98%) believe that besides instruments, disinfecting the dental chair, clinic, and doctor's office is necessary. In Table 3, it has been observed that there is a significant positive correlation between the mean score of knowledge and attitude *r* = 0.781, *P* ≤ 0.05; however, there was no differences noted between the mean score of attitude-practice and knowledge-practice scores.

## 4. Discussion

Hands are the foremost significant reservoir for many pathogens. Handwashing is therefore considered as an effective method of prevention of infection [15]. In the current study, the majority of the students described that hands should be washed and the students described plain soap and antiseptic solution before and after the examination as a preferred method. Similar results were seen in a study conducted in Germany where most of the trainee dentists washed their hands before and after the procedure [8]. Nonetheless, there are studies that show poor compliance of the students with handwashing [9]. A study conducted in Yemen reported only 43% of the students adhere to handwashing [9]. In the present study, most of the dental students were vaccinated with hepatitis B vaccine which was in concurrence with the other studies [12–14]. This rate is much higher than the study conducted in India showing that only 38% of the students were vaccinated against hepatitis B [6]. The dental students had good knowledge of autoclaving and sterilization similar to other studies [6, 9]. Wearing of face mask and eye protection was observed in only one-third of the students. Similar results were recorded in other studies [9]. However, a good majority of the dental students in our study thought that hepatitis B infection and not tuberculosis is transmitted through saliva, which was quite surprising. Another surprising finding in our study was that two-thirds of the students considered that ineffective sterilization during clinical practice cannot transmit infection from one patient to another. This highlights the lack of awareness of the concept of cross infection. The majority of the students did believe that disinfection of dental chairs and the office of the dentist is also required. This concurs with the findings of other studies included in our literature review [2, 3]. Importance of continuous-based infection control lectures and training could help in raising the level of knowledge regarding the subject [16]. We recommend that every hospital needs to set up infection control measures in accordance with the guidelines. For both the students and faculty of a hospital to improve their practice and knowledge of infection control, hospitals need to address the need for quality assurance and implementation of infection control measures. It is highly recommended that reinforcement training of the students through periodic workshops on infection precaution and control should be planned to highlight the significance of infection control [17]. Another recommendation is that of vaccination, especially hepatitis B, which should be mandatory for all medical and dental students prior to their admission.

The strength of this study is that it was conducted with a senior group of dental students (3^rd^ and 4^th^ year) who have studied the module on infection prevention and control. The use of a self-administered questionnaire itself is one of the limitations of this study; therefore, objectivity in responding to certain questions might have been compromised. Results could be generalized with caution because similar group of students in a private hospital setting with better infection control and prevention standard operating procedures might exhibit different behaviors and practices.

## 5. Conclusion

We concluded that there was lack of correct knowledge on infection control among the dental students, yet they showed a positive attitude towards infection control measures. However, a greater compliance and supervision would be needed. Further studies in similar settings and utilizing the mix methods with qualitative enquiry would be beneficial to understand certain careless attitudes and behaviors among dental students.

## Figures and Tables

**Table 1:** Gender distribution and average score of knowledge, attitude, and practice of dental students in each group.

Gender	3rd year	4th year	*P* value
Male (28 (14.9%))	12 (12.0%)	16 (18.2%)	0.235
Female (160 (85.1%))	88 (88.0%)	72 (81.8%)	
Knowledge	10.80 ± 2.50	10.07 ± 1.81	0.024
Attitude	4.70 ± 1.32	4.45 ± 1.36	0.212
Practice	5.32 ± 1.22	5.86 ± 1.20	0.003

**Table 2:** Student knowledge, attitudes, and practice on infection control measures.

			*n* = 188	%
1	Do you wash your hands before and after patient examination?	Yes	176	93.6

2	With what do you wash your hands?	Plain soap	88	46.8
		Detergent	8	43
		Antiseptic solution	92	48.9

3	Do you prefer oral mouth rinse before commencement of any treatment procedure?		120	63.8

4	Do you think isolation is important in infection control?		184	97.9

5	With which of the following vaccines have you been vaccinated?	Hepatitis B	160	85.1
		Tetanus	84	44.7
		Tuberculosis	28	14.9
		None	34	18.1

6	Which of the following do you use to sterilize instruments in dental clinic?	Autoclave	178	94.7
		Boiling	8	4.3
		Washing	2	1.1

7	Minimum time required for sterilization in autoclave?	5 min	44	23.4
		10 min	16	8.5
		15 min	128	68.1

8	Temperature for sterilization in autoclave?	100 degree	62	33
		120 degree	112	59.6
		150 degree	14	7.4

9	Which of the following has the highest rate of transmission via saliva?	Hepatitis B	150	79.8
		AIDS	12	6.4
		Tuberculosis	14	7.4
		Do not know	12	6.4

10	What immediate action should be taken in case of direct blood contact with HIV patient?	Anti-HIV immunoglobulins	116	61.7
		Anti-HIV drugs	22	11.7
		Blood test to be carried out	48	25.5
		Do not know	2	1.1

11	What protective measures do you take to prevent yourself from injury?	Face mask and gloves	72	38.3
		Eyewear	60	31.9
		Protective clothing	2	1.1
		All the above	54	28.7

12	After use of gloves for a patient, what do you do with them?	Dispose them	108	57.4
		Reuse them after wash	80	42.6
		Reuse them after sterilization	0	0

13	Ineffective sterilization during clinical practice can transmit infection from one patient to another.		70	37.2

14	Apart from instrument sterilization, disinfection of dental chair, clinic, and dental office is required?	Yes	184	97.9
		Do not know	2	1.1

**Table 3:** Correlation among knowledge, attitudes, and practice scores.

Variables	Correlation coefficient (*r*)	*P* value
Knowledge-attitude	0.781	0.000
Attitude-practice	-0.157	0.031
Knowledge-practice	-0.045	0.539

## Data Availability

Available with first author.
